# The relationship of social support to posttraumatic growth in COVID-19 among college students after experiencing campus lockdown: the effects of belief in a just world and meaning in life

**DOI:** 10.3389/fpsyt.2024.1337030

**Published:** 2024-01-25

**Authors:** Aoyu Wu

**Affiliations:** Department of Civil Engineering, Hebei Jiaotong Vocational and Technical College, Shijiazhuang, China

**Keywords:** COVID-19, campus lockdown, social support, belief in a just world, meaning in life, PTG

## Abstract

**Background:**

Campus lockdown orders were issued for the purpose of preventing and controlling COVID-19, which resulted in psychological problems among college students. However, the experiences they have during the pandemic may also lead to positive personal changes, including posttraumatic growth (PTG). The current study examined the mediating role of belief in a just world and meaning in life in social support and PTG during the COVID-19 campus lockdown.

**Method:**

An online survey was conducted on 1711 college students in Hebei Province, China. Based on the survey results, a structural equation model was established.

**Results:**

Social support positively predicted PTG. Furthermore, belief in a just world and meaning in life played a mediating role between social support and PTG respectively. Besides, social support could also predict PTG through the multiple serial mediating effect of belief in a just world and meaning in life.

**Conclusion:**

These results indicated mechanisms by which social support influenced PTG, and this provided insights into how to promote post-traumatic growth among university students in the post-pandemic period.

## Introduction

The global outbreak of COVID-19 in 2020 has had a major impact on people’s lives, not only threatening the physical health of individuals, but also impacting people’s mental health (1–6). The COVID-19 is considered as a new type of collective trauma (7). Research found that insomnia and posttraumatic stress symptoms are very common among college students, 31.33% and 16.36% of college students are found having depression and anxiety symptoms (8). Changes in living environment and learning patterns, unable to participate in social activities normally, and especially having COVID-19-related viral panic and contagious fear about the epidemic have impacted the mental health of college students greatly (9, 10). In addition to causing negative psychological effects on individuals, traumatic events may also lead to positive changes in their worldview, values, attitudes towards themselves and others, leading to posttraumatic growth (PTG) (11), which is a positive psychological change experienced by individuals after fighting against traumatic events (12). Many researches are currently focused on the negative effects of the pandemic on mental health, however, in the post-pandemic era, it is very relevant for researchers to turn their attention to the potential growth opportunities presented by adversity. Moreover, for the sake of college students’ health and well-being, lockdown orders were issued to prevent the epidemic on campus at the beginning of the Fall semester of 2022 in China. Little research is done in the context of campus lockdown, considering the distinctiveness of this policy, it is necessary to conduct research on college students who have experienced campus lockdowns. During the pandemic, whether it is the destruction caused by the epidemic in media reports or the experience of infection, individuals are more likely to reflect on themselves actively, e.g., interpersonal relationships, work, and life status, and think about the meaning of life, which will stimulate the occurrence of posttraumatic growth.

“What doesn’t kill you makes you stronger”, thus spoke Nietzsche. Seizing opportunities in times of crisis and promoting growth is a desirable vision for people, but turning danger into opportunity requires certain conditions. Posttraumatic growth (PTG) refers to the positive psychological changes that individuals experience after experiencing traumatic situations or events (12). The Theory of Crises and Personal Growth (13) believes that environmental factors are the key affecting whether individuals could gain growth or not after trauma, and social support, which refers to the material and spiritual assistance a person gained from social relationships such as family, relatives, friends, and organizations (14), is an important environmental factor. The Model of Thriving Through Relationships (15) believes that the reason for individuals to experience Posttraumatic Growth is due to having a strong social support system. Good interpersonal relationships can provide individuals with a safe atmosphere, facilitate the expression of negative emotions, and enable them to be accepted and comforted promptly, which is beneficial for individuals to devote more energy to coping with crisis events, encouraging individuals to actively think about traumatic events and reduce psychological distress. Therefore, this theory believes that social support can not only promote individuals to discover their potentials to cope with difficulties, but also help them reflect on crisis events and construct their cognition actively, discover the meaning behind traumatic events, and achieve post-traumatic growth. Besides, from the perspective of Resource Conservation Theory (16), social support is a social resource owned by individuals that provides material security and enables individuals to obtain psychological support when under pressure, thereby promoting posttraumatic growth. Numerous studies have shown that social support can predict posttraumatic growth significantly (17–19). On this basis, this study proposes H1: Social support positively predicts posttraumatic growth among college students.

The purpose of the present study is to explore the mechanism of social support on PTG in the context of COVID-19 epidemic by establishing a structural equation model to examine the mediating role of belief in a just world and meaning in life in the relationship between social support and PTG.

### The mediating role of belief in a just world

The theory of belief in a just world holds that people need to believe that the world they live in is stable and orderly, with predictable outcomes, and that they will be treated fairly without becoming victims of unforeseeable disasters. This provides people with a sense of security and control. Only under this premise can people have confidence in the future and pursue long-term goals with the belief that they will eventually get what they deserve (20). Therefore, researchers consider belief in a just world to be an important psychological resource. During the COVID-19 epidemic, quarantine became the most common coping measure, and social deprivation would affect individuals’ belief in a just world significantly (21). At this time, effective social support could provide individuals with a safe environment and coping resources (19, 22–24), and buffer the psychological impact of the epidemic, increasing individuals’ belief in a just world level (18, 25, 26). Individuals with high belief in a just world have strong emotional regulation abilities (21, 27), which helps to maintain a good level of mental health and improve well-being in life. According to the Posttraumatic Growth Model (12), an individual’s perception of the world is an important predictor of posttraumatic growth. After experiencing disaster events, individuals’ beliefs in a just world often change (28). Individuals with high belief in a just world tend to have higher levels of trust in interpersonal and social organizational relationships mostly and have a more positive attitude towards the future (29). Positive perceptions of the world can increase confidence in the face of injustice (27), and buffer the negative effects of traumatic events, which promotes posttraumatic growth (28). Therefore, we hypothesize that the belief in a just world may have a mediating effect between social support and posttraumatic growth accordingly. On this basis, this study proposes H2: Belief in a just world plays a mediating role between social support and posttraumatic growth among college students.

### The mediating role of meaning in life

Meaning in life refers to an individual’s perception and awareness of human beings and the nature of their existence, as well as those things that they consider important, including the two dimensions of presence of meaning and search for meaning (30). According to the Shattered Assumptions Theory of Posttraumatic Growth (31) traumatic events can shatter assumptions about oneself and the world, making it necessary to rethink the meaning of life. When the meaning in life is reconstructed, individuals would form a new understanding of themselves and the world, and the new hypothesis towards the world begins to emerge. After experiencing a traumatic event, support and encouragement from others are important sources of meaning in life (32). Social support helps to reconstruct the meaning of traumatic events, and view adversity from a positive perspective, through which individuals could explore psychological resources to cope with difficulties and establish a basic understanding of themselves and the world. What’s more, new life goals that individuals striving for will be set (33). Research has shown that social support provides a warm and receptive psychological atmosphere that allows people to confide in others when they are in pain, thereby promoting their sense of meaning and hope facing adversity (34). Therefore, social support is positively correlated with meaning in life significantly (32, 35). Moreover, individuals who experience the meaning of life in the face of difficulties are more likely to experience posttraumatic growth (36–39). In view with the above, we hypothesize that social support may influence posttraumatic growth through meaning in life. On this basis, this study proposes H3: Meaning in life plays a mediating role between social support and posttraumatic growth among college students.

### The multiple serial mediating role of belief in a just world and meaning in life

An effective social support system provides people with a safe psychological atmosphere, alleviates psychological impact when facing difficulties, and helps maintain a high belief in a just world. According to the Meaning-Making Model (40), beliefs constitute the core schema interpreting life experiences which are an important foundation to develop unique and stable life meaning experiences for individuals. Beliefs in a just world can guide people to conduct more positive self-evaluation and provide internal motivations to pursue long-term goals, helping them experience a higher sense of meaning in life (41) (42). When individuals witness or experience severe injustice, such that they are unable to maintain their belief in a just world, they may believe that the world is fundamentally random, and nothing goes around comes around. This may lead them to give up on long-term goals and seek immediate short-term goals, or simply giving them all up (20), reducing the sense of meaning in life. In addition, meaning of life enables individuals to recognize the value of their own existence, have a higher sense of self-efficacy, maintain a positive attitude in the face of difficulties (43), and strive to achieve goals in adversity. These positive changes in worldview, values, and attitudes about self and interpersonal relationships may indicate one’s posttraumatic growth in the face of adversity. Therefore, we can infer that social support may have a positive effect on the belief in a just world when facing difficulties, and individuals with a higher belief in a just world have a higher sense of life meaning by rethinking themselves and the world, which is conducive to the formation of posttraumatic growth. On this basis, this study proposes H4: Belief in a just world and meaning in life mediate the relationship between social support and posttraumatic growth among college students through their chain mediating effect.

**Figure 1:**
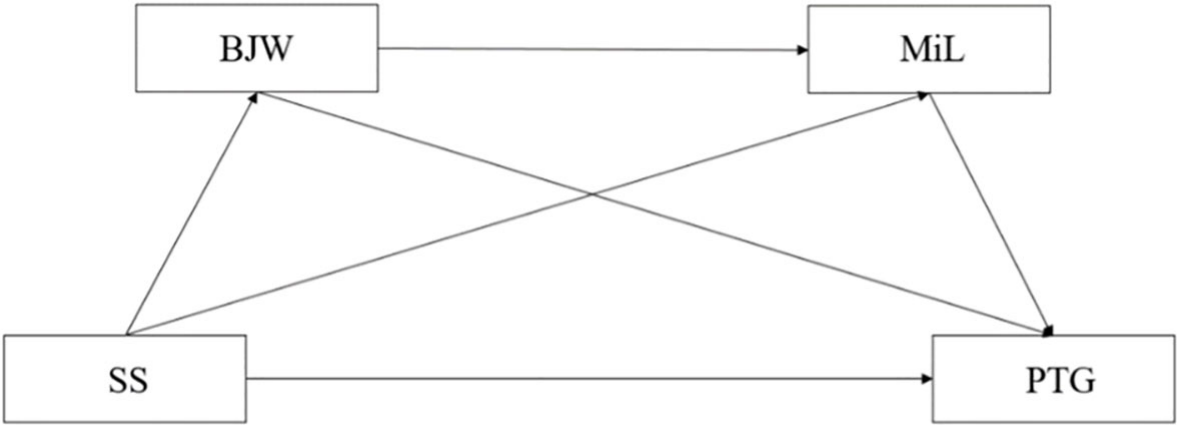
Conceptual model; SS, social support; BJW, belief in a just world; MiL, meaning in life.

To sum up, this study proposes the following hypothesis, and the conceptual model is presented in **Figure 1**:

H1: Social support positively predicts posttraumatic growth among college students.
H2: Belief in a just world plays a mediating role between social support and posttraumatic growth among college students.
H3: Meaning in life plays a mediating role between social support and posttraumatic growth among college students.
H4: Belief in a just world and meaning in life mediate the relationship between social support and posttraumatic growth among college students through their chain mediating effect.

## Method

### Participants

An online questionnaire survey was conducted on 1711 college students (mean age = 19.89 years old, *SD* = 2.36, range = 18-24 years old), among which 1047 males (61.2%) and 664 females (38.8%) participated, including undergraduate and vocational colleges with 51 majors offered, in Hebei Province between March 7th and 15th, 2023. There were 847 freshmen (51.1%), 697 sophomores (40.7%), 73 juniors (4.3%), and 67 seniors (4.0%) participated in the survey. It is worth mentioning that the students were all quarantined at school during the fall semester of 2022.

### Measures

#### Perceived social support scale

The Perceived Social Support Scale (PSSS) was compiled by Zimet et al. (44) and the Chinese version was revised by Jiang Qianjin (45). The revised scale consists of 12 items arising from three groups, namely family, friend, and significant others. Participants answered these using a 7-point Likert scale ranging from 1 (completely disagree) to 7 (completely agree). The total score reflects the overall level of social support perceived by the individual, and greater score indicates a higher level of perceived social support. The Cronbach’s alpha was 0.96 in the present study. Factor loading of every item is > 0.4, and AMOS was used to conducted Confirmatory Factor Analysis (CFA) on the questionnaire and the results showed that the model fit is well, indicating the validity of the tools is acceptable: *χ²/df* = 1.92, CFI = 0.91, TLI = 0.92, RMSEA = 0.069, SRMR = 0.06. The original data was organized in Stata/MP13.1, and average variance extracted (AVE) and composite reliability (CR) are calculated using Excel 2013 (46). AVE and CR on family support are 0.49 and 0.7, AVE and CR on friend support are 0.55 and 0.83, and AVE and CR on other support are 0.48 and 0.79, indicating the validity of the tools is acceptable.

#### Belief in a just world scale

Belief in a just world scale developed by Zhou Chunyan (20) was used. The scale consists of 17 items, including two lie detection items, which were rated on a 6-point Likert scale ranging from 1 (completely disagree) to 6 (completely agree). The total score reflects the level of belief in a just world, and greater score indicates a higher level of belief in a just world. The Cronbach’s alpha for the total scale was 0.94 in the present study. Factor loading of every item is > 0.4, and AMOS was used to conducted CFA on the questionnaire and the results showed that the model fit is well, indicating the validity of the tools is acceptable: *χ²/df* = 2.05, CFI = 0.91, TLI = 0.90, RMSEA = 0.075, SRMR = 0.08. The original data was organized in Stata/MP13.1, and average variance extracted (AVE) and composite reliability (CR) are calculated using Excel 2013 (46). AVE and CR on self-present are 0.44 and 0.67, AVE and CR on others-future are 0.45 and 0.67, AVE and CR on other-present are 0.36 and 0.61, and AVE and CR on self-future are 0.47 and 0.78 respectively, indicating the validity of the tools is acceptable.

### Meaning in life questionnaire

The present study used the Meaning in Life Questionnaire (MLQ) compiled by Steger, Frazier, Oishi, and Kaler (30) and translated by Liu and Gan (47). The questionnaire consists of two subscales: the search for meaning and the presence of meaning with a total of nine items. Participants respond to the items on a 7-point Likert scale ranging from 1 (absolutely untrue) to 7 (absolutely true). Greater score indicates higher presence and search, and the Cronbach’s alpha was 0.86 for the total scale in the current study. Factor loading of every item is > 0.4, and AMOS was used to conducted CFA on the questionnaire and the results showed that the model fit is well, indicating the validity of the tools is acceptable: *χ²/df* = 1.91, CFI = 0.92, TLI = 0.91, RMSEA = 0.069, SRMR = 0.06. AVE and CR on search for meaning are 0.45 and 0.78, and AVE and CR on presence of meaning are 0.51 and 0.79 respectively, indicating the validity of the tools is acceptable.

### Posttraumatic growth inventory

PTG was assessed using the Posttraumatic Growth Inventory (PTGI) developed by Tedeschi and Calhoun (11) and revised by Wang and Wu (48). There are a total of 22 items, including five subscales, namely personal strength, new possibilities, relating to others, appreciation of life, and spiritual change. It was scored on a 6-point Likert scale from 0 (no change) to 5 (experienced this change to a very great degree). The Cronbach’s alpha was 0.97 for this scale in our sample. Factor loading of every item is > 0.4, and AMOS was used to conducted CFA on the questionnaire and the results showed that the model fit is well, indicating the validity of the tools is acceptable: *χ²/df* = 1.94, CFI = 0.95, TLI = 0.93, RMSEA = 0.06, SRMR = 0.06. AVE and CR on personal strength are 0.51 and 0.79 respectively, AVE and CR on new possibilities are 0.47 and 0.78 respectively, AVE and CR on relating to others are 0.45 and 0.76 respectively, AVE and CR on appreciation of life are 0.55 and 0.83 respectively, AVE and CR spiritual change are 0.49 and 0.79 respectively, indicating the validity of the tools is acceptable.

### Procedure and date analysis

The statistical analyses were performed using SPSS 24.0 and Amos 24.0. Firstly, we used Harman’s single factor test to determine if there is a common method bias (49). Factor analysis was conducted on all items, and a total of 7 factors with eigenvalues > 1 were selected. The variation explained by the first factor was 30.2%, less than the critical value of 40%, indicating that there was no significant common method bias in this study. Afterwards, descriptive and correlation analysis were conducted. On this basis, a structural equation modeling method (50) was used to examine the mediating role of belief in a just world and meaning in life between social support and posttraumatic growth, after controlling gender, place of origin, and income as covariates.

## Results

### Discrepancy test and correlation analysis of various variables

The results show that there was no significant difference in grade and whether the only child or not among the variables. **Tables 1**, **2** show the differences in gender, origin, and income level among the variables. From **Table 1**, the gender difference in the belief in a just world and post traumatic growth is significant, with female students having significantly higher scores in the belief in a just world than male students; and the posttraumatic growth score of males is significantly higher than that of females. In addition, there were significant differences in social support, belief in a just world, and sense of life significance among students from different regions of origin. College students from urban areas scored significantly higher on these three variables than college students from rural areas. There are significant differences in social support, belief in a just world, and meaning in life at the income level. Students from middle-income families have significantly higher scores in social support, belief in a just world, and meaning in life than students from low and high income families.

**Table 1:** Analysis of differences in gender and origin among various variables.

	Gender			Origin		
	Male	Female		Urban	Rural	
	(*n*=1047)	(*n*=664)	*t*	(*n*=434)	(*n*=1277)	*t*
SS	5.23 ± 1.68	5.23 ± 1.08	0.89	5.38 ± 1.17	5.18 ± 1.12	3.13^***^
BJW	4.11 ± 0.98	4.25 ± 0.76	3.33^**^	4.24 ± 0.92	4.13 ± 0.89	2.18^*^
MiL	4.98 ± 1.10	4.89 ± 0.97	1.71	5.04 ± 1.08	4.92 ± 1.04	2.15^*^
PTG	4.25 ± 1.02	4.12 ± 0.95	2.56^**^	4.22 ± 1.04	4.20 ± 0.98	0.32

^*^p<0.05,^**^p<0.01,^***^p<0.001; SS, social support; BJW, belief in a just world; MiL, meaning in life.

**Table 2:** Analysis of differences in income levels among various variables.

	Income levels			
	Low-income (*n*=1369)	Middle-income (*n*=277)	High-income (*n*=65)	F
SS	5.18 ± 1.12	5.44 ± 1.14	5.38 ± 1.30	6.57^**^
BJW	4.12 ± 0.89	4.33 ± 0.89	4.17 ± 1.51	5.95^**^
MiL	4.91 ± 1.03	5.12 ± 1.04	4.92 ± 1.39	4.38^**^
PTG	4.18 ± 0.98	4.31 ± 0.96	4.28 ± 1.30	2.01

^**^p<0.01; SS, social support; BJW, belief in a just world; MiL, meaning in life.

**Table 3** shows the mean scores, standard deviations, and correlation matrices for each variable. The Pearson correlation analysis indicates that social support, belief in a just world, meaning in life, and posttraumatic growth are positively inter-correlated among one another.

**Table 3:** Descriptive results and correlation analysis between variables.

Variables	M	SD	1	2	3	4
1. SS	5.23	1.14				
2. BJW	4.16	0.90	0.63^**^			
3. MiL	4.95	1.05	0.47^**^	0.55^**^		
4. PTG	4.20	0.99	0.43^**^	0.46^**^	0.43^**^	

n=1711; ^**^p<0.01; SS, social support; BJW, belief in a just world; MiL, meaning in life.

### Testing for the mediation effects

**Figure 2:**
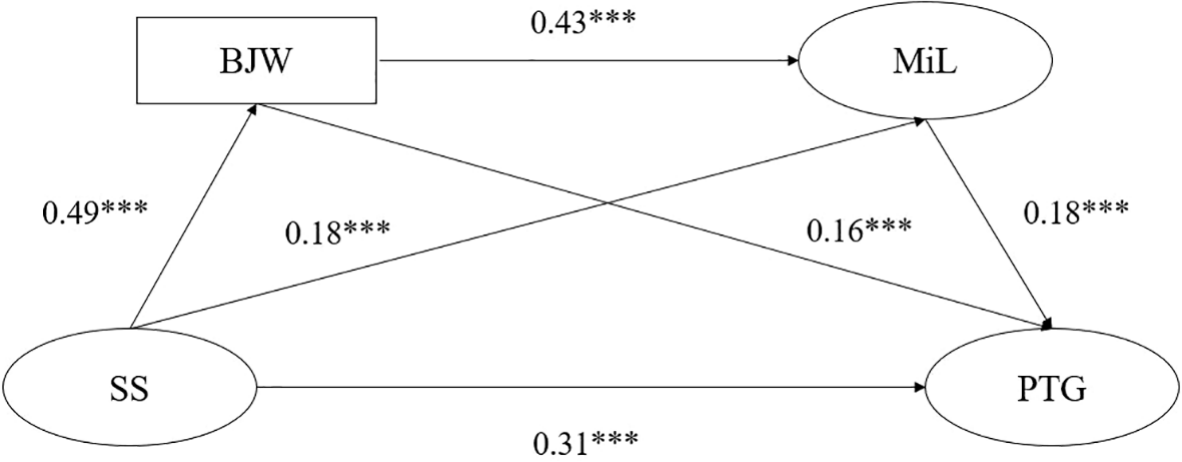
Proposed mediation model. ***p<0.001.

The PROCESS v4.2 macro program was used for the mediation analysis, repeated sampling 5000 times from the original date to calculate the 95% CI. If the 95% CI of the standardized path coefficient does not contain 0, it indicates that the mediating effect is significant (51). The results show that after controlling for gender, place of origin, and income level, the overall fit model fit is within an acceptable range (*χ^2^/df* = 8.63, CFI = 0.98,TLI = 0.97, RMSEA = 0.067), and the model is presented in **Figure 2**.

The results show that the effect of social support on PTG was significant (direct effect = 0.31, 95% CI: 0.267, 0.356), while that on belief in a just world and meaning in life were significant mediators between social support and PTG (total indirect effect = 0.15, 95% CI: 0.111, 0.194). And the mediating effect is partial. To be specific, belief in a just world played a mediating role between social support and PTG (indirect effect = 0.08; 95% CI: 0.040, 0.114); meaning in life played a mediating role between social support and PTG (indirect effect = 0.03, 95% CI: 0.019, 0.048); belief in a just world and meaning in life played a serial mediating role between social support and PTG (indirect effect = 0.04, 95% CI: 0.028, 0.060). None of the bootstrapped 95% CI includes zero, confirming the mediating effect of belief in a just world and meaning in life. Furthermore, in the relationship between social support and PTG, the mediating effect of belief in a just world was significantly greater than that of meaning in life (the mediating effect of belief in a just world minus the mediating effect of meaning in life = 0.05; 95% CI: 0.003, 0.100). These results illustrate that belief in a just world and meaning in life play a mediating role between social support and PTG. Moreover, compared to meaning in life, belief in a just world had a more substantial relation on social support to PTG of college students after COVID-19 campus lockdown.

## Discussion

The purpose of the present study is to explore the mechanism of social support on PTG in the context of COVID-19 epidemic by establishing a structural equation model to examine the mediating role of belief in a just world and meaning in life in the relationship between social support and PTG.

The results of the study showed that social support could positively predict PTG, which was consistent with previous studies (18, 19, 25). From the perspective of the Model of Thriving through Relationships, social support, as an important personal resource, provides individuals with a safe atmosphere and creates an environment to alleviate negative emotions facing difficulties, which is beneficial to view the current adversity from a positive perspective, thereby promoting the development of PTG. The study further investigated the mediating effect between social support and PTG and found that both the belief in a just world and meaning in life can play a mediating role.

We found that social support could promote post-traumatic growth by positively predicting the belief in a just world, which was consistent with previous research (18, 52–54). As a public health emergency of international concern, the COVID-19 disrupted people’s routines. Continuous quarantine and isolation have reduced the sense of control and security that individuals felt. As for college students in their youth who cannot have a normal campus life, the deprivation of interpersonal relations reduced their belief in a just world. To prevent the epidemic on campus, China had adopted measures of lockdowns even in campus without outbreaks. This measure had protected the safety of staff’s lives, but it had also harmed the psychological well-being of students to some extent (55). Strict lockdowns prevented college students from engaging in normal social interactions with people outside of their own school (56), and they may feel treated unjustly. People were unable to control their own lives. The sense of absurdity when order was broken could easily make people feel that life is meaningless, and their sense of meaning was reduced (57). Afterwards, China had adjusted its policies in a timely manner based on the development of COVID-19, and no longer imposed strict lockdowns on campus. Staff were able to come in and go out of schools normally. The restoration of social interaction and life order allowed people to rebuild meaning in life, which facilitate PTG (19). Nowadays, in the post-pandemic era, the order of life is gradually being restored and college students value interpersonal relationships even more (58). With a good social support system, college students begin to reconstruct their understanding of the world. Moreover, normal social interaction makes college students feel supported and cared for by others, which helps to maintain and improve the quality of existing interpersonal relationships, thereby obtaining a higher sense of social support (59). To sum up, in warm and receptive psychological environment like this, the psychological impact of the epidemic on college students can be alleviated, which helps to rebuild belief in a just world. The positive understanding of the world can promote the development of PTG. This result validates the Posttraumatic Growth Model (12).

Moreover, the study showed that social support could promote PTG by enhancing meaning in life of college students. Specifically, encouragement and support from others are important sources for individuals to have higher level of meaning in life when experiencing difficulties. Previous studies have also found that maintaining the meaning in life in adversity helps individuals face challenges with a more positive mindset. During the pandemic, meaning in life felt by college students played a crucial role in their psychological growth. According to the Shattered Assumptions Theory of Posttraumatic Growth, the things that happened pandemic may break college students’ original understanding of the world, causing them to lose the sense of control over themselves, others, or the world, and even the meaning in life as a result (60). In the post pandemic era, normal social interactions enable college students to feel that they have more social support. And the warm interpersonal environment can divert their attention from past negative experiences, encouraging individuals to pay attention to positive information. In this way, college students gradually regain a sense of control over their lives, so they set and strive for new goals, which will reshape their meaning in life. Besides, the meaning in life can also help college students discover the positive connotations behind disasters, which enables them to gain hope and face life with a grateful attitude. From the past experiences, college students learn to explore their potential when facing difficulties, thereby achieving PTG.

Our results also confirmed that social support played a positive role in predicting PTG through a chain mediating effect of belief in a just world and meaning in life. In the post-pandemic period, college students with high social support experience more psychological warmth, providing a safe environment and coping resources, which enables them to have a high belief in a just world even in a disordered life. The strength that college students leads them to recognize and reassess the difficult situation they are facing actively, through which helps them construct meaning in life, and see adversity as an opportunity to stimulate their own growth, namely PTG.

In all, through this study, we found that the impact of social support on PTG can be achieved through the belief in a just world and meaning of life. The research integrated the Model of Thriving Through Relationships (15) and the Shatter Assumptions Theory (31) effectively, which has enlightening significance for the study of the mechanism of PTG. In addition, this study also provided some inspiration for the well-being of college students after the epidemic. To promote PTG of college students who have experienced adversity in real life, social support should be provided to individuals. For example, during school lockdown and quarantine, school staff should provide enough help to college students. And timely attention ought to be paid to college students with prominent emotional fluctuations. In particular, staff need to help students establish trust in themselves and the world and make them realize the meaning of life to avoid losing faith in life during adversity. What’s more, this study suggests that except for focusing on alleviating negative psychological problems among college students affected by the epidemic, a positive belief in the world should be established as well. To maintain the meaning in life and people’s well-being, the policy makers should take people’s need for the belief in a just world into account. To achieve PTG, college students ought to master the ability to explore the strength faced with problems and learn to overcome difficulties.

However, this study also has some limitations. This study adopted a cross-sectional study design and cannot discuss the changes and causal relationships between variables over time. Some researchers believe that PTG can be individual’ coping strategies when facing traumatic events, which may fade after a period (61). So future research can follow-up this group to further confirm incidence of PTG and the long-term mechanism of social support on it. Besides, compared to other countries, China’s policies had their own distinctive characteristics, so the experiences of Chinese college students during the epidemic is unique as well, and it is necessary to verify the model’s generalizability in cross-cultural scenarios.

## Conclusions

Through the research on the relationship between social support, belief in a just world, meaning in life and posttraumatic growth of college students who had experienced school lockdown during COVID-19 pandemic, the following conclusions are drawn: Social support can not only directly promote PTG, but also through belief in a just world and the meaning in life. It can also be promoted through the serial mediation effect of belief in a just world and meaning in life.

## Data availability statement

The raw data supporting the conclusions of this article will be made available by the authors, without undue reservation.

## Ethics statement

The studies involving humans were approved by The ethics committee of Hebei Jiaotong Vocational and Technical College. The studies were conducted in accordance with the local legislation and institutional requirements. The participants provided their written informed consent to participate in this study. Written informed consent was obtained from the individual(s) for the publication of any potentially identifiable images or data included in this article.

## Author contributions

AW: Conceptualization, Data curation, Formal Analysis, Investigation, Methodology, Project administration, Resources, Software, Supervision, Validation, Visualization, Writing – original draft, Writing – review & editing.
